# Acute Toxicity and No Observable Adverse Effect Level of Nodularin-R in Mice

**DOI:** 10.3390/toxins18080336

**Published:** 2026-08-01

**Authors:** Sarah C. Finch, Craig Waugh, D. Tim Harwood, Alison R. Turnbull, Andrew I. Selwood, Emillie M. F. Passfield, Jonathan Puddick

**Affiliations:** 1 AgResearch Group—Bioeconomy Science Institute, Ruakura Research Centre, Private Bag 3123, Hamilton 3240, New Zealand; 2 Cawthron Institute, Private Bag 2, Nelson 7042, New Zealand; craig.waugh@cawthron.org.nz (C.W.); tim.harwood@cawthron.org.nz (D.T.H.); andy.selwood@cawthron.org.nz (A.I.S.); emillie.passfield@cawthron.org.nz (E.M.F.P.); 3 New Zealand Food Safety Science and Research Centre, Massey University, Private Bag 11 222, Palmerston North 4442, New Zealand; 4 Institute for Marine and Antarctic Studies, University of Tasmania, 15-21 Nubeena Crescent, Tasmania 7053, Australia; alison.turnbull@utas.edu.au

**Keywords:** cyanobacteria, food safety risk assessments, nodularin-R, *Nodularia spumigena*, toxicity

## Abstract

Nodularin is a cyanobacterial toxin predominantly produced by *Nodularia spumigena* Mertens. This cyanobacterium has been associated with fish, bird, and animal deaths, and the toxin can contaminate fish and shellfish intended for human consumption. With climate change, blooms of *Nodularia spumigena* are becoming more frequent and more widespread, meaning that the risk this cyanobacterium poses to human health must be evaluated. However, toxicity information is scarce, meaning that data from the closely related toxin class, microcystins, are currently used instead. In the current study, the LD_50_ of nodularin-R by intraperitoneal injection was found to be 50 µg/kg, consistent with previous reports. For the first time, the LD_50_ of nodularin-R by oral administration was determined using the voluntary consumption of laced cream cheese (7.5 mg/kg), which is similar to that reported for microcystin-LR (10.9 mg/kg). The use of microcystin toxicology data in food safety assessments for nodularin, therefore, seems reasonable—but it is critical that an oral repeated-dose study is performed on nodularin to better understand the risk posed by this toxin through chronic exposure.

## 1. Introduction

**Figure 1:**
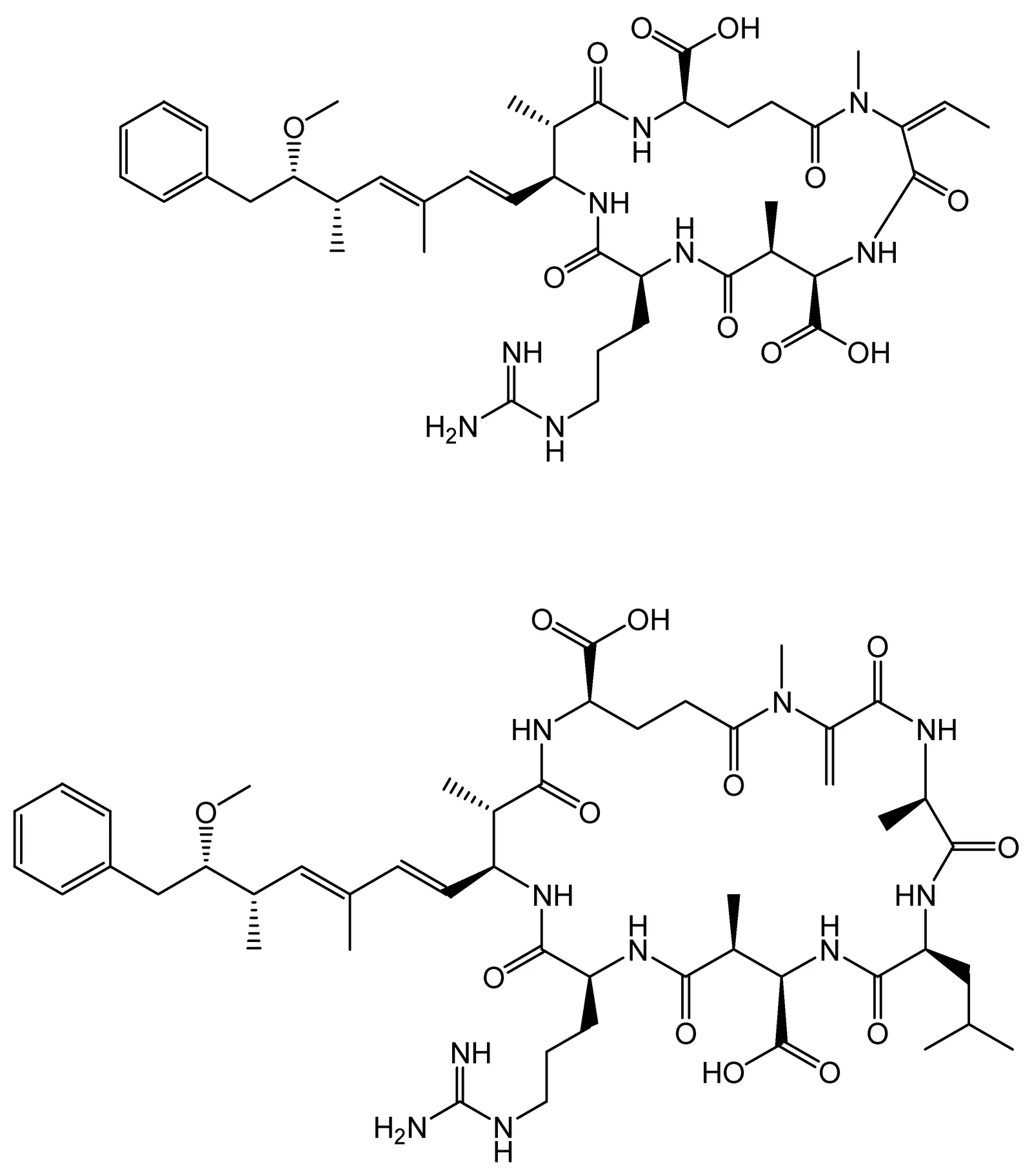
Chemical structures of nodularin-R (**top**) and microcystin-LR (**bottom**).

Nodularin is a cyanobacterial toxin predominantly produced by the blue-green algae *Nodularia spumigena* Mertens, but has also been reported in *Iningainema pulvinus* [1], *Nodularia sphaerocarpa* [2], several *Nostoc* species [3,4] and *Nostochopsis* sp [5]. This compound, first recognized in 1988 [6,7], is structurally related to the microcystin class of compounds. The microcystin class is composed of over 300 different structural variants [8] whereas the nodularin class comprises only 10 structural variants which are characterized by having a smaller ring system. Both classes of toxin accumulate in the liver and act upon protein phosphatases, resulting in liver damage and altered liver functioning [9,10]. For each toxin class the most common structural congeners present in the environment are microcystin-LR and nodularin-R (Figure 1).

*Nodularia spumigena* is most commonly found in brackish and estuarine waters over the summer months and was first reported as an issue in 1878. In this case, horses, sheep, pigs and dogs that ate the cyanobacterial material from Lake Alexandrina in Australia [11] died within 1–8 h of consumption [12]. Since this time, reports linking *Nodularia spumigena* to the death of fish, birds, and domestic and wild animals have occurred throughout the world. Dog deaths appear to be most common, due to ingestion of toxin-containing cyanobacteria from the licking of fur following exposure to blooms in water bodies. Dog deaths have been documented at the German North Sea coast [13], from the Baltic Sea [14] and in South Africa [10]. Furthermore, livestock deaths have been reported in Australia [15], the UK [16] and New Zealand [17].

In addition to animal deaths, nodularin can contaminate shellfish, fish and crustaceans. Nodularin has been detected in mussels from western Australia [18], seafood originating from the Gippsland Lakes area, Australia (prawns, mussels and finfish) [19], mussels and fish caught in the Baltic Sea [20,21], and eels in New Zealand [17]. Since nodularin is present in seafood, this compound can move up the food chain and be eaten by humans. For example, nodularin has been detected in Eiders, seabirds in Finland, that feed on contaminated mussels and which form part of the diet of humans [21]. Human exposure to nodularin is associated with diarrhea, vomiting, weakness, anorexia and muscle tremors [20]. However, unlike microcystin, which was associated with the death of 76 dialysis patients exposed to contaminated water used in the preparation of intravenous treatments in Brazil [22], nodularin has not been associated with human mortality.

Due to climate change, the presence of *Nodularia spumigena* is increasing and blooms now occur frequently in many areas including the Baltic sea [20], Australia [23] and New Zealand [17], reigniting interest in nodularin. Although values for the median lethal dose (LD_50_) of nodularin-R determined in mice by intraperitoneal (i.p) injection have been published [24,25], other toxicity data on this compound are scarce. Since the current nodularin toxicity data are insufficient for regulatory purposes, data determined for microcystins have been used to assess the potential risk of nodularins to human health [26]. This is deemed appropriate because of the structural similarities, shared mode of action and similar acute toxicity by i.p administration [19].

In this study we determine the LD_50_ of nodularin-R to mice by i.p injection. In addition, for the first time, the acute oral toxicity of nodularin-R was determined, as well as a no observable adverse effect level (NOAEL), using voluntary feeding. These data allow comparison with published data for microcystins to assess the validity of using microcystin data in the assessment of the human health risk to nodularins.

## 2. Results

### 2.1. Isolation of Nodularin-R

Nodularin-R was successfully isolated from freeze-dried environmental bloom material. The purified nodularin-R material was dried to produce a white solid weighting 55.8 mg. HPLC-UVD quantitation of the nodularin-R in methanol indicated a concentration of 12.4 mg/mL giving a total of 51.1 mg nodularin-R and ~1 mg 6*Z*-Adda-nodularin-R. Gravimetric purity was determined at 96.5% based on the nodularin existing as a formate salt. The quantity of 6Z-Adda-nodularin is likely to be slightly underestimated using the HPLC-UVD method due to the difference in absorbance maxima of 3 nm compared with nodularin-R (Supporting Information Figure S1). LC-MS scanning showed nodularin-R as 97.4% of the material, with 6*Z*-Adda-nodularin-R as the only detected impurity at 2.6%. NMR spectroscopy analysis closely agreed with LC-MS purity with 97.6% and 2.4% for nodularin-R and 6*Z*-adda-nodularin-R respectively (Table 1). 6*Z*-Adda-nodularin-R has no toxicity [27]. Product-ion spectra were consistent with reported nodularin fragmentation, and NMR data conclusively identified the compound as nodularin-R [28] (Supporting Information Figures S3 and S4, and Tables S1 and S2).

### 2.2. Acute Toxicity of Nodularin-R by Intraperitoneal Injection

The median lethal dose (LD_50_) of nodularin-R by i.p injection was 50 µg/kg (95% confidence intervals 43.7–66.9 µg/kg). Individual mice were dosed at 40 µg/kg (female, lived but showed a hunched posture and orbital tightening which persisted for 1 h post-dosing), 50 µg/kg (female, died, euthanized due to severe symptoms 4.5 h post-dosing), 40 µg/kg (female, lived, normal throughout), 50 µg/kg (female, lived but showed clinical signs of toxicity as described above for 4.5 h post-dosing), 63 µg/kg (female, died, discomfort displayed with ears back, head down, hunched posture and orbital tightening, slowing respiration was also displayed and the mouse was euthanized 1 h post-dosing), 50 µg/kg (female, lived, but showed discomfort for 2 h post-dosing and low food intake over the first day), 63 µg/kg (male, died, clinical signs of toxicity as described above, euthanized 1 h post-dosing). All mice that survived the nodularin-R dosing were observed for 14 days. Mice were checked daily and all showed normal appearance, behavior, food intake and weight gain on days 2–14. At necropsy no abnormalities were observed and the major organs, expressed as a percentage of body weight, were within normal limits.

### 2.3. Acute Toxicity of Nodularin-R by Oral Administration

The median lethal dose (LD_50_) of nodularin-R by oral administration using the voluntary consumption of toxin-laced cream cheese was 7.5 mg/kg (95% confidence intervals 1.5–8.8 mg/kg). Female mice were used throughout and all nodularin-R laced cream cheese doses were consumed completely within 30 s. Individual mice were dosed at 10 mg/kg (died, at 4.5 h post-dosing the mouse looked uncomfortable with eyes closed and a hunched posture, at 6 h post-dosing the mouse was prostrate and was euthanized), 7.5 mg/kg (lived, at 5 h post-dosing the mouse had a hunched posture and food intake was low over the first day), 10 mg/kg (died, at 2.5 h post-dosing the mouse was observed with its head down and ears back, at 3.5 h post-dosing the mouse was prone and it was euthanized), 7.5 mg/kg (died, at 3 h post-dosing ears were back, which persisted until 5 h post-dosing when movement was wobbly, at 5.5 h post-dosing movement was uncoordinated and the mouse was euthanized), 5 mg/kg (lived, showed a hunched posture at 4 h post-dosing and food intake was low over the first day), 7.5 mg/kg (died, at 3.75 h post-dosing the mouse had its ears back and head was down, at 4.5 h post-dosing eyes were closed and at 5.5 h post-dosing it was euthanized). All mice that survived the nodularin-R dosing were observed for 14 days. Mice were checked daily and all showed normal appearance, behavior, food intake and weight gain on days 2–14. No abnormalities were seen at necropsy and the major organs, expressed as a percentage of body weight, were within normal limits.

### 2.4. No Observable Adverse Effect Level of Nodularin-R by Oral Administration

To determine the NOAEL of nodularin-R by oral administration using the voluntary consumption of toxin-laced cream cheese, groups of five mice (four female and one male) were dosed at 2.5, 3.5 and 5 mg/kg. Of the mice dosed 5 mg/kg, three showed mild clinical signs and one showed a low food intake over the first 24 h. Of the mice dosed 3.5 mg/kg, two showed minor clinical signs and one showed a low food intake over the first 24 h. None of the five mice dosed at 2.5 mg/kg showed any clinical signs and food intake was normal over the 14-day observational period. No abnormalities were seen at necropsy in any of the treatment groups and the major organs, expressed as a percentage of body weight, were within normal limits. The NOAEL for nodularin-R by oral administration using the voluntary consumption of laced cream cheese is therefore 2.5 mg/kg.

## 3. Discussion

Nodularin-R was successfully isolated from *Nodularia spumigena* bloom material with a purity of 97%. The contaminant, 6Z-Adda-nodularin-R is known to be non-toxic [27]. The LD_50_ determined for nodularin-R, in this work, by i.p administration was similar to that of previous reports (Table 2). The LD_50_ was also similar to that of microcystin-LR especially when accounting for the different molecular weights and expressing on a molar basis (Table 2).

This is the first report of the oral toxicity of nodularin-R with the LD_50_ being slightly lower than the published value for microcystin-LR (Table 3). It is also worth noting that the microcystin data were generated using gavage dosing, which is known to overestimate the toxicity of shellfish toxins [30]. This is because, unlike a human stomach, the contents of a mouse stomach is paste-like, allowing the liquid gavage dose to flow around the semi-solid mass to be rapidly absorbed by the duodenum. The voluntary consumption of a toxin-laced solid matrix by mice, such as the cream cheese used in this study, avoids this issue.

Although the acute oral toxicities of nodularin-R and microcystin-LR are similar, people do not always consume only one meal of contaminated food but rather can eat multiple meals. For this reason, the most important data for use in the calculation of regulatory limits come from sub-acute or sub-chronic dosing. The data reported in this paper do not raise any major concern for the use of microcystin toxicology data in the assessment of food safety concerns related to nodularin. However, it is crucial that a repeated dose study be performed to provide more robust data and allow a more accurate determination of risk. Since nodularin-R is a hepatotoxin, it is important that a possible effect on the liver is included in this assessment through serum chemistry and liver histology. Due to a possible effect of gender, it is also important that this future study has treatment groups of both genders.

## 4. Materials and Methods

### 4.1. Purification of Nodularin-R

#### 4.1.1. Extraction of Crude Nodularin Material

Freeze-dried wild-harvested cyanobacterial biomass from Wairewa/Lake Forsyth (37 g, Container Nod-4) was suspended in Milli-Q water (700 mL) and sonicated for 15 min at 59 kHz to disrupt cells in an ultrasonic bath (KUDOS, Shanghai, China). This was then centrifuged at 4000× *g* for 5 min and the supernatant collected. Extraction of the cyanobacterial pellet was repeated three more times with 600 mL Milli-Q water per extraction. Combined extracts were acidified with 5% (*v*/*v*) glacial acetic acid by slow addition with stirring and refrigerated for 24 h to precipitate proteinaceous co-extractives, then centrifuged at 4000× *g* for 5 min.

#### 4.1.2. Solid-Phase Purification and Desalting

The clarified extract was applied to a 100 × 25 mm column packed with C_18_ sorbent (30 g; United Chemical Technologies, Bristol, PA, USA), preconditioned with acetonitrile followed by Milli-Q water. Nodularin was eluted using a linear gradient from 0.1% acetic acid in water to 0.1% acetic acid in acetonitrile over 40 min at 25 mL/min (Sepacore X50; BUCHI, Flawil, Switzerland). Fractions collected were assessed by LC-MS/MS, and nodularin-containing fractions pooled. Acetonitrile was removed by rotary evaporation. The concentrate was loaded onto a weak cation exchange cartridge (30 g Sepra ZT-WCX; Phenomenex, Torrance, CA, USA) and eluted using a linear gradient from 0.1% acetic acid in 50% acetonitrile to 0.5 M acetic acid in 50% acetonitrile over 40 min at 25 mL/min. Nodularin-positive fractions were pooled and acetonitrile removed by rotary evaporation. To exchange solvents, the pooled material was adsorbed onto a 30 g C_18_ cartridge, washed with 100 mL Milli-Q water, then eluted with 100% methanol. Fractions of 1 × 20 mL and 2 × 40 mL were collected and analyzed by LC-MS/MS; the first 40 mL fraction (Fraction 2) contained nodularin and was retained.

#### 4.1.3. Preparative HPLC

To separate the 6*Z*-Adda-nodularin-R isomer from nodularin-R, preparative HPLC was performed (Prominence LC20AP; Shimadzu, Kyoto, Japan). The nodularin material was dissolved in 50% methanol to give a final concentration of approximately 10 mg/mL. A volume of 500 µL was injected onto a Gemini C_18_ column (20 × 150 mm; Phenomenex, USA) using a linear gradient from 20% mobile phase B to 100% mobile phase B over 1 min, then held for 9 min (where mobile phase A was 0.1% formic acid in water and mobile phase B was 0.1% formic acid in 60% methanol). Elution was monitored at 238 nm using a photodiode array detector. Nodularin-R and 6*Z*-Adda-nodularin-R peaks were collected in separate fractions. The system was re-equilibrated to initial conditions between injections. Pooled nodularin-R fractions were evaporated *in vacuo*.

#### 4.1.4. Analytical Characterization of Purified Nodularin-R

**Gravimetric preparation and dilution:** The dried nodularin-R material was weighed on a five-decimal-place balance to determine the mass present. Methanol (3.25 g) was added to dissolve the compound, then a 1000-fold dilution into methanol was prepared for analysis.

**HPLC with UV detection:** Quantitation and purity were determined by HPLC-UVD (Prominence LC-20AD; Shimadzu, Kyoto, Japan) using a Gemini C_18_ column (2.1 × 150 mm; Phenomenex, USA). Isocratic elution was performed with 55% methanol containing 0.1% formic acid at 0.5 mL/min for 10 min. Detection was performed at 238 nm. External calibration used a nodularin-R certified reference material from the National Research Centre Canada (NRCC), and the relative contribution of nodularin-R and 6*Z*-Adda-nodularin-R was expressed as percentage area at 238 nm.

**LC-MS:** LC-MS analyses used the same chromatographic conditions as HPLC-UVD. Detection was performed on an 8050 mass spectrometer (Shimadzu, Kyoto, Japan), employing positive-mode electrospray ionization scanning 400–1200 Da, with collision-induced dissociation at 35 eV to fragment nodularin.

**NMR spectroscopy:** Purified nodularin-R (1.2 mg) was dissolved in 600 µL CD_3_OD (99.8+ atom-% D; Aldrich, St. Louis, MO, USA). NMR (^1^H and HSQC) spectra were acquired using a Bruker NEO 500 MHz NMR spectrometer (Bruker BioSpin, Ettlingen, Germany), with a 5 mm inverse TCI cryoprobe. Chemical shifts were referenced to internal CHD_2_OD at 3.31 ppm and CD_3_OD at 49.0 ppm. The data was processed using MestReNova V15.0 (Mestrelab Research, Spain). The ^1^H spectrum matched the published nodularin spectrum, and both ^1^H and ^13^C chemical shifts agreed with literature values.

### 4.2. Animals

Male and female Swiss albino mice (18–22 g) used in the study were bred at the Bioeconomy Science Institute (AgResearch Group, Hamilton, New Zealand). Mice were kept in a temperature-controlled room (21 ± 1 °C) with a 12 h light–dark cycle and with unrestricted access to food (Rat and Mouse Cubes; Specialty Feeds Ltd., Glen Forrest, Australia) and water. All work was approved by the AgResearch Animal Ethics Committee established under Animal Welfare Act, 1999 (New Zealand; Project Number 1983, approval date 30 May 2024).

### 4.3. Toxicity Determination of Nodularin-R

Acute toxicity was determined according to the principles of the Organization for Economic Co-operation and Development (OECD) guideline 425 [31]. This guideline uses the lowest animal numbers while still allowing a robust determination of the LD_50_, including an estimate of confidence intervals. This method uses an up-and-down protocol whereby an individual animal is given a dose rate at one step below the estimated LD_50_. If this animal survives, the dose rate given to the next animal is increased by a factor determined by the computer program associated with this method made by estimating the slope of the dose–response curve. If the first animal dies, the next animal is given a dose rate decreased by the same factor. This process continues until four live-death reversals have been achieved. To avoid any diurnal effect in response, all dosing was conducted between 8:00 and 9:30 a.m. All mice were observed closely during the day of dosing. Survivors were monitored for a 14-day period including regular measurement of body weight and food intake. After 14 days, the animals were killed using carbon dioxide inhalation and necropsied. Prior to dosing mice were weighed and test compounds dosed on a µg/kg basis.

For toxicity by i.p injection aliquots of the methanolic solution were taken and diluted with 1% Tween/saline (250 µL/mouse). For the determination of toxicity by oral administration mice were first trained to consume cream cheese. This was achieved by offering groups of 4 mice small amounts of cream cheese twice daily. Once the cream cheese was happily consumed the mice were individually caged and cream cheese (150 mg) was offered on a watch glass. If this amount was consumed within 30 s the mice were deemed ready for the experiment and if not, further training was necessary. For dosing, aliquots of the nodularin-R solution were mixed with cream cheese (150 mg).

### 4.4. No Observable Adverse Effect Level of Nodularin-R

An NOAEL was determined by feeding groups of five mice doses of nodularin-R incorporated into cream cheese as described above. Food intake and body weight was measured regularly and after 14 days, the animals were euthanized using carbon dioxide inhalation and necropsied. Major organs were weighed and expressed as percentage of body weight.

## Figures and Tables

**Table 1:** Summary of nodularin purity as determined by each analytical method.

Analytical Method	Nodularin-R	6*Z*-Adda-Nodularin-R
Gravimetric	96.5%	N/A
HPLC-UVD	98.1%	1.9%
LC-MS	97.4%	2.6%
NMR	97.6%	2.4%

**Table 2:** Acute toxicities (LD_50_) determined by intraperitoneal injection for nodularin-R (NOD-R) and microcystin-LR (MC-LR).

Compound	µg/kg	nmol/kg	Reference
NOD-R	50 (43.7–66.9) *	60.6 (53.0–81.1) *	This work
NOD-R	50	60.6	Eriksson et al., 1988 [24]
NOD-R	70	84.8	Runnegar et al., 1988 [25]
MC-LR	65.4	65.7	Yoshida et al., 1997 [29]

* Numbers in parenthesis denote 95% confidence intervals.

**Table 3:** Acute toxicities (LD_50_) determined by oral administration for nodularin-R (NOD-R) and microcystin-LR (MC-LR).

Compound	mg/kg	µmol/kg	Reference
NOD-R	7.5 (1.5–8.8) *	9.1 (1.8–10.7) *	This work
MC-LR	10.9	11.0	Yoshida et al., 1997 [29]

* Numbers in parenthesis denote 95% confidence intervals.

## Data Availability

The original contributions presented in this study are included in the article/Supplementary Material. Further inquiries can be directed to the corresponding author(s).

## Supplementary Materials

The following supporting information can be downloaded at: https://www.mdpi.com/article/10.3390/toxins18080336/s1, Figure S1: HPLC-UVD chromatogram (238 nm) of the purified nodularin-R showing peaks integrated for nodularin-R (1) and 6Z-Adda-nodularin-R (2). Inserts of UV spectra for the two compounds are also shown, with a lambda max of 237 nm for nodularin and 240 nm for 6Z-Adda-nodularin-R; Figure S2: Total ion chromatogram for the purified nodularin acquired by positive ESI scanning; Figure S3: LC-MS positive ion mass spectrum of the purified nodularin-R (A) and product-ion spectrum of the [M+H]^+^ adduct ion (B); Figure S4: NMR ^1^H spectrum of CNC00165 (top) and published ^1^H spectrum of nodularin-R (bottom) [28]; Figure S5: Structures of nodularin-R and the modified Adda moiety of 6Z-Adda-nodularin-R, with the proposed MS/MS cleavage sites indicated; Table S1: NMR chemical shifts (ppm) of nodularin-R reported by Namikoshi et al. [28] and for the material purified in this study (lot CNC00165); Table S2: ^1^H chemical shifts for protons in the C-6 region of the minor isomer in CNC00165 compared to reference chemical shifts for nodularin-R and 6Z-Adda-nodularin-R.

## Abbreviations

i.p	Intraperitoneal
LD_50_	Median lethal dose
MC-LR	Microcystin-LR
NOAEL	No observable adverse effect level
NOD-R	Nodularin-R
