# Supplementary material for: Acute Toxicity and No Observable Adverse Effect Level of Nodularin-R in Mice
Source: Toxins (Basel). 2026 Aug 1;18(8):336. doi: 10.3390/toxins18080336 (PMC13517166; doi:10.3390/toxins18080336)
Supplement: Supplementary file 1 [file toxins-18-00336-s001.zip › toxins-4371604-supplementary.pdf]

Supplementary Materials: Acute Toxicity and No Observable Adverse Effect Level of Nodularin-R in Mice

Sarah C. Finch, Craig Waugh, D. Tim Harwood, Alison R. Turnbull, Andrew I. Selwood, Emillie M. F. Passfield and Jonathan Puddick

The isolated nodularin-R was assayed for quantity by HPLC-UVD (Figure S1). The purity and identity were confirmed by LC-MS and NMR spectroscopy. The purified compound was conclusively identified as nodularin using NMR spectroscopy. The ^{1}H spectrum matched the published spectrum (Figure S4, [28]). Both ^{1}H and ^{13}C chemical shifts match published values (Table S1). Low levels of the 6Z-Adda isomer were detected in the ^{1}H spectrum. The identity of this isomer was confirmed by comparing ^{1}H chemical shifts of protons in the region of the isomerization (C-6) to reported values [28]. The relative concentration of the 6Z-Adda isomer is estimated to be 2.4% using the ^{1}H integral of the H-5 proton. This closely matched the concentration determined by LC-MS.

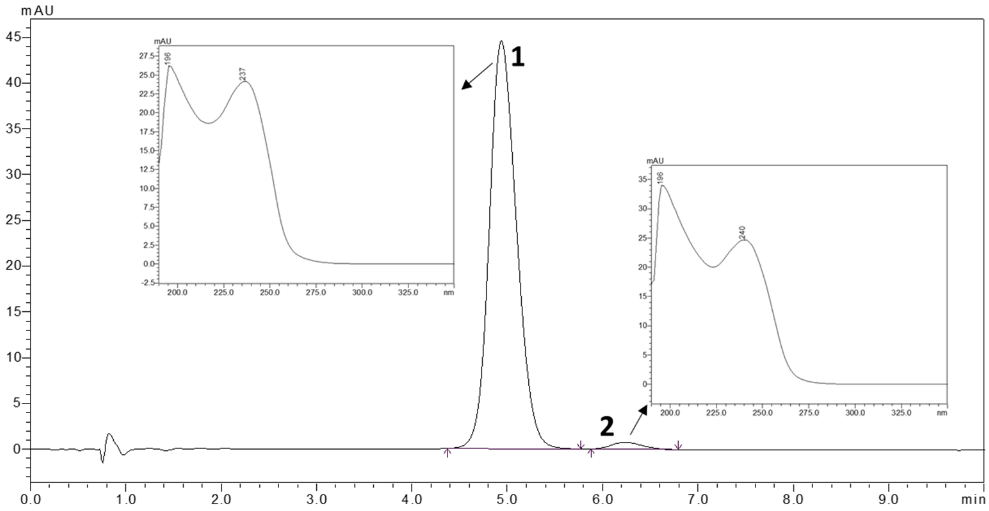

Figure S1. HPLC-UVD chromatogram (238 nm) of the purified nodularin-R showing peaks integrated for nodularin-R (1) and 6Z-Adda-nodularin-R (2). Inserts of UV spectra for the two compounds are also shown, with a lambda max of 237 nm for nodularin and 240 nm for 6Z-Adda-nodularin-R.

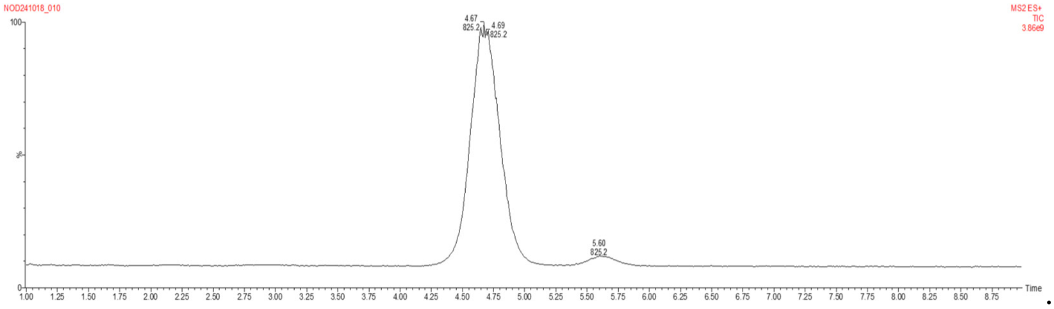

Figure S2. Total ion chromatogram for the purified nodularin acquired by positive ESI scanning.

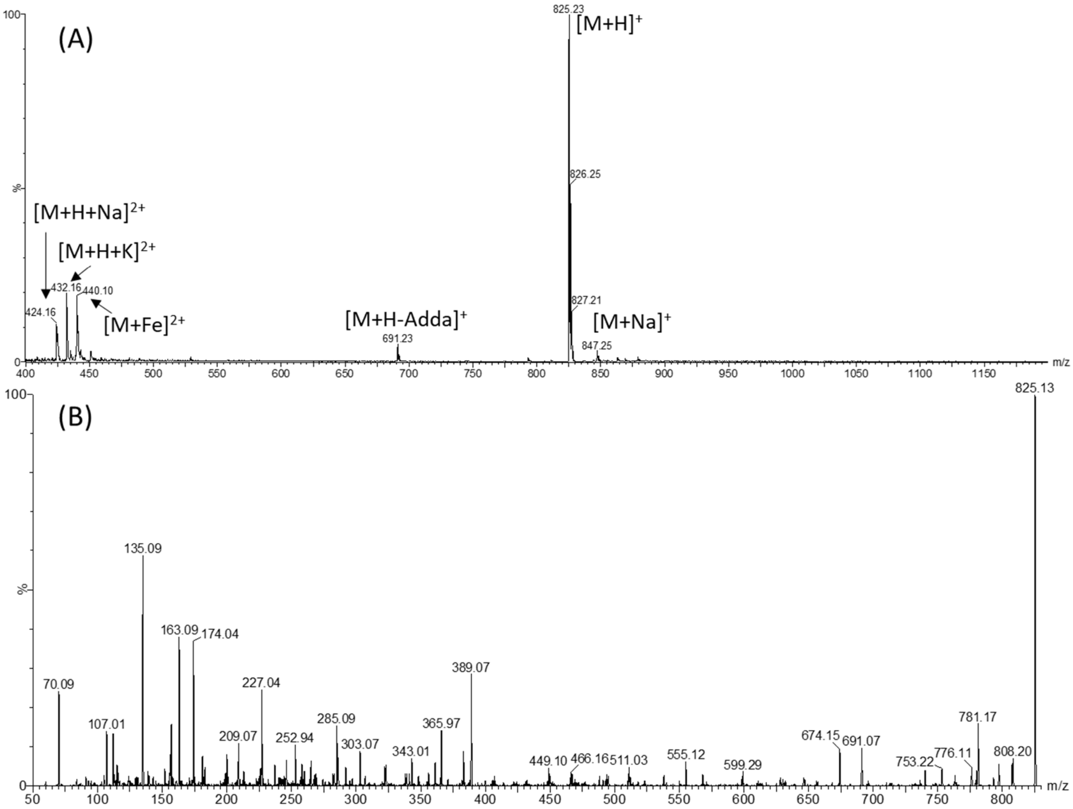

Figure S3. LC-MS positive ion mass spectrum of the purified nodularin-R (A) and product-ion spectrum of the $[M+H]^+$ adduct ion (B).

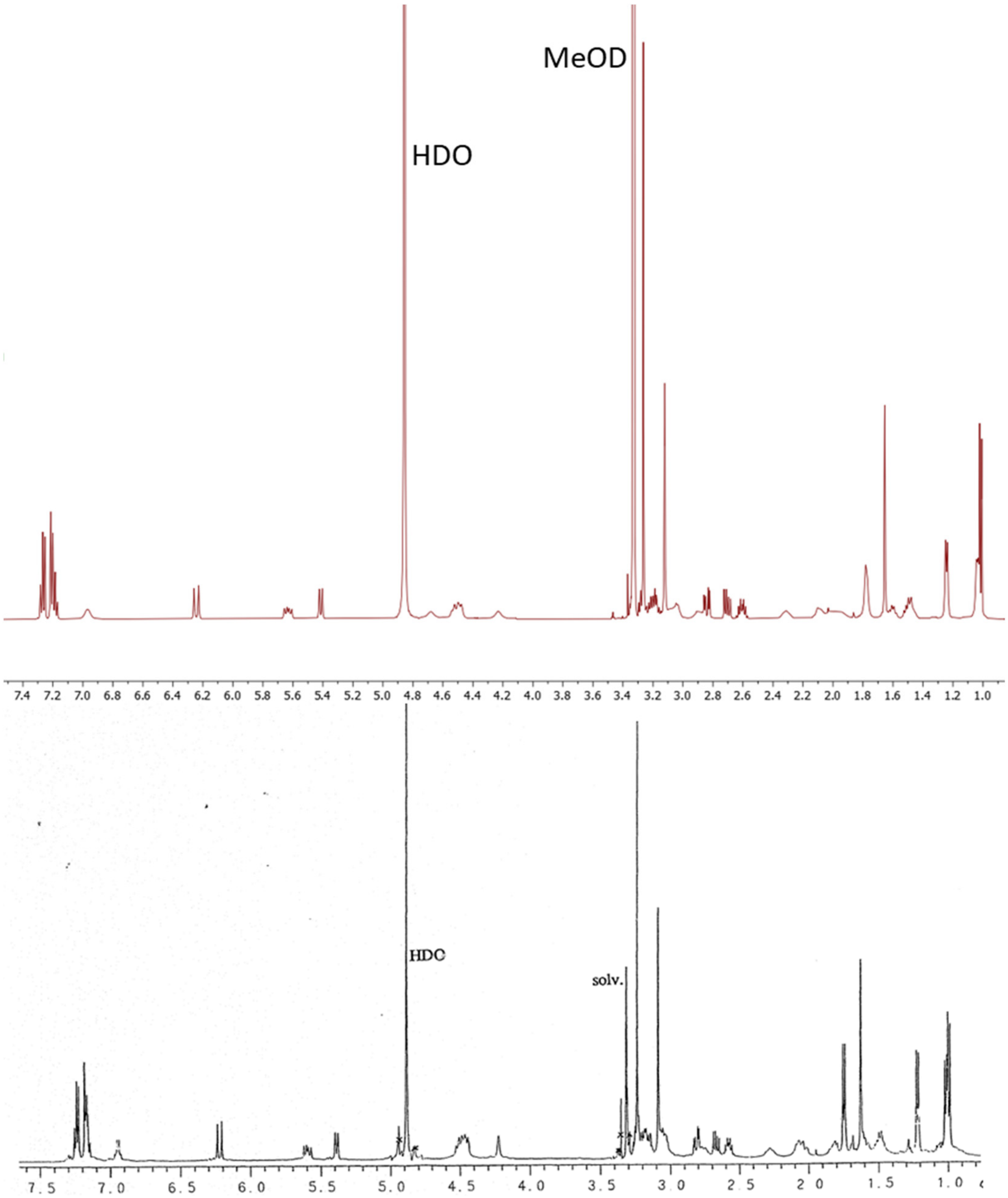

Figure S4. NMR ^{1}H spectrum of CNC00165 (top) and published ^{1}H spectrum of nodularin-R (bottom) [28].

Figure S5. Structures of nodularin-R and the modified Adda moiety of 6Z-Adda-nodularin-R, with the proposed MS/MS cleavage sites indicated.

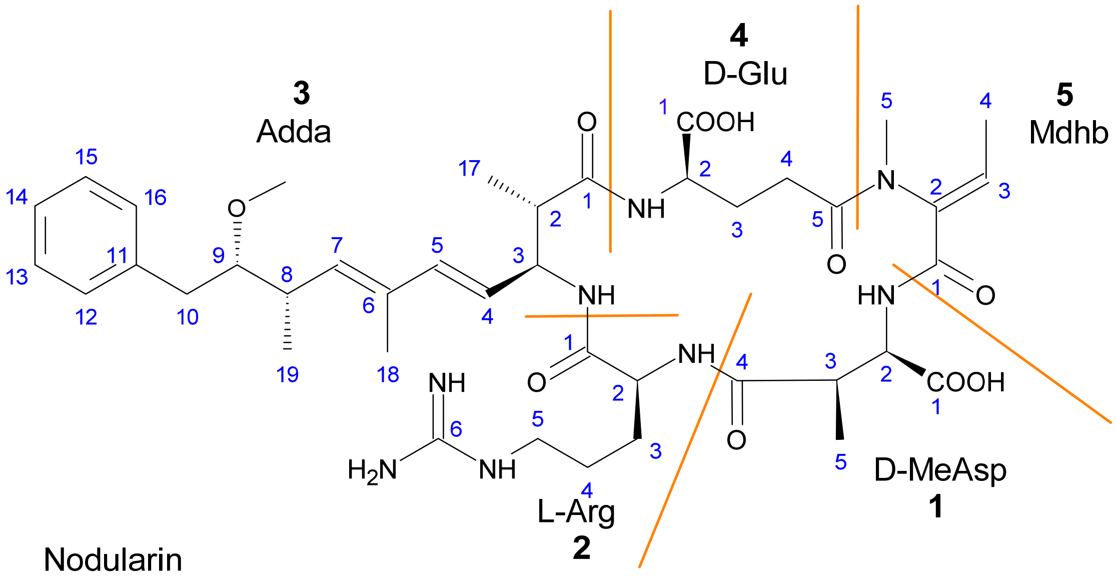

Nodularin

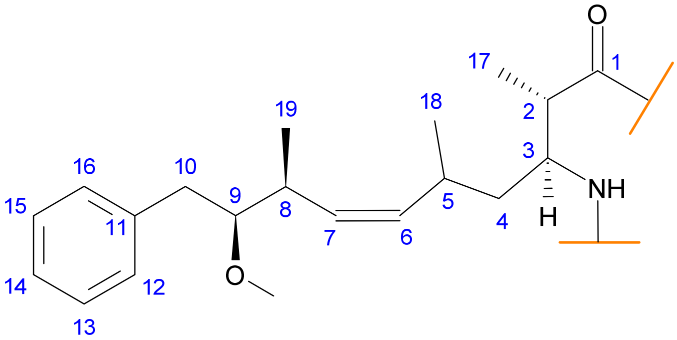

6Z-Adda-nodularin

Table S1. NMR chemical shifts (ppm) of nodularin-R reported by Namikoshi et al. [28] and for the material purified in this study (lot CNC00165).

	Namikoshi et al 1994		Cawthron (CNC00165)	
	**1H**	**13C**	**1H**	**13C**
**[MeAsp]**				
1		177.9		ND
2	4.22	60.5	4.21	60.1
3	3.04	41.1	3.02	41.2
4		178.3		ND
5	1.21	16.3	1.22	16
**[Arg]**				
1		172		ND
2	4.45	52	4.46	51.3
3	1.47, 2.07	29.2	1.45, 2.08	28.9
4	1.49, 1.61	25.9	1.48, 1.58	25.4
5	3.18	41.9	3.18	41.5
6		158.6		ND
**[Adda]**				
1		177		ND
2	3.03	44.7	3.06	44.1
3	4.5	56.7	4.5	56.5
4	5.6	126.8	5.61	126.7
5	6.21	139	6.22	138.8
6		134.2		ND
7	5.38	136.8	5.39	136.4
8	2.58	37.7	2.59	37.4
9	3.24	88.4	3.25	88.1
10	2.66, 2.81	39	2.69, 2.82	38.6
11		140.5		ND
12,16	7.17	130.5	7.19	130.2
13,15	7.24	129.2	7.25	128.9
14	7.16	127	7.17	126.7
17	1.02	16.4	1.02	15.6
18	1.62	13	1.63	12.7
19	0.98	16.6	1	16.2
20	3.23	58.7	3.16	58.6
**[Glu]**				
1		176.4		
2	4.46	54.5	4.65	51.7

3	1.8, 2.26	29	1.93, 2.29	27.8
4	2.05, 2.79	29.9	2, 2.87	29
5		175.6		
**[Mdhb]**				
1		165.6		ND
2		137.8		ND
3	6.94	136.6	6.94	136.3
4	1.74	13.3	1.76	12.9
5	3.08	35.2	3.1	34.7

.

ND = Not determined as quaternary carbon chemical shifts are not detected in an HSQC spectrum.

Table S2. ^{1}H chemical shifts for protons in the C-6 region of the minor isomer in CNC00165 compared to reference chemical shifts for nodularin-R and 6Z-Adda-nodularin-R.

	Namikoshi et al 1994		Cawthron CNC00165
	**Nodularin-R**	**6Z-Adda-nodularin-R**	**Minor isomer**
**H-5**	6.21	6.5	6.5
**H-4**	5.6	5.75	5.72
**H-7**	5.38	5.25	5.27
**H-10**	2.66, 2.81	2.63, 2.74	2.63, 2.76
**H-18**	1.62	1.84	1.82
